# Monthly Record of Deaths in the City of Buffalo

**Published:** 1859-03

**Authors:** P. H. Strong

**Affiliations:** Health Physician


ART. VIII.—*Report of Mortality in Buffalo for the Month of Jan.*, 1859.
By P. H. Strong, M. D., Health Physician.

| DISEASES. | | No. | Males. | Females. | No Sex given. |
|---|---|---|---|---|---|
| Abscess of Liver, | | 1 | 1 | | |
| Accidental, | | | | | |
| By Burning Fluid, | 1 | | | | |
| By Drowning, | 1 | | | | |
| By a Falling Tree, | 1 | | | | |
| By Falling down Stairs, | 1 | | | | |
| Poisoned by Opium, | 1 | 5 | 3 | 2 | |
| Anæmia, | | 1 | 1 | | |
| Apoplexy, | | 3 | 2 | 1 | |
| Acute Bronchitis, | | 1 | 1 | | |
| Cholic, (Painter's,) | | 1 | 1 | | |
| Cancer, | | 1 | | 1 | |
| Congestion of Lung, | | 2 | 1 | | |
| Congestion of Brain, | | 2 | 1 | 1 | |
| Consumption, (of which 7 were certified by undertakers,) | | 15 | 7 | 8 | |
| Convulsions, Infantile, (of which 8 were certified as (fits) by undertakers,) | | 15 | 10 | 5 | |
| Croup, | | 6 | 3 | 3 | |
| Debility, Infantile, | | 3 | 2 | | 1 |
| Delirium Tremens, | | 1 | 1 | | |
| Diarrhœa, Infantile, | | 2 | | 2 | |
| Dropsy, | | 2 | | 2 | |
| Dropsy of Pericardium, | | 1 | | 1 | |
| Dropsy in the Brain, | | 3 | 3 | | |
| Dysentery, | | 1 | | 1 | |
| Fever, Congestive, | | 1 | 1 | | |
| " Inflammatory, | | 1 | 1 | | |
| " Puerperal, | | 1 | | 1 | |
| " Scarlet, | | 5 | 3 | 2 | |
| " Typhoid, | | 2 | 1 | 1 | |
| Fistula in Perineum, | | 1 | 1 | | |
| Frost Bitten, | | 2 | 1 | 1 | |
| Heart, Disease of, | | 3 | 2 | 1 | |
| Hæmorrhagica Purpura, | | 1 | | 1 | |
| Hooping Cough, | | 2 | 1 | 1 | |
| Hypertrophy of Liver and Spleen, | | 1 | 1 | | |
| Inflammation of Brain | | 4 | 2 | 2 | |
| Inflammation of Bowels, | | 1 | 1 | | |
| Inflammation of Lungs, | | 5 | 3 | 2 | |
| Inflammation of Stomach, Chronic, | | 3 | 1 | 2 | |
| Intemperance, | | 1 | | 1 | |
| Marasmus, Infantile, | | 2 | 1 | 1 | |
| Old Age, | | 5 | 1 | 4 | |
| Ovarian Tumor, | | 1 | | 1 | |
| Paralysis, | | 1 | 1 | | |
| Pericarditis, | | 1 | | 1 | |
| Peritonitis, | | 1 | 1 | | |
| Premature Birth, | | 1 | | 1 | |
| Pyemia, | | 1 | 1 | | |
| Rheumatism, | | 2 | 1 | 1 | |
| Still Born, | | 3 | 2 | | 1 |
| Stomatitis Materua, | | 1 | | 1 | |

REGISTER OF MORTALITY—CONTINUED.

| DISEASES. | No. | Males. | Females. | No Sex given. |
|---|---|---|---|---|
| Small Pox, | 3 | 3 | | |
| Suicide by Hanging, | 1 | 1 | | |
| Unknown, | 5 | 4 | 1 | |
| Total, | 128 | 73 | 53 | 1 |

| | |
|---|---|
| Of this number there were certified by Undertakers, | 30 |
| " " " " by Coroner, | 9 |

| | |
|---|---|
| Died at the Almshouse, | 5 |
| " Hospital of the Sisters of Charity, | 4 |
| " Foundling Asylum, | |

SEXES.

| | |
|---|---|
| Males, | 73 |
| Females, | 53 |
| Sex not given, | 2 |
| Total, | 128 |

AGES.

| | | | |
|---|---|---|---|
| Still-born, | 3 | Between 20 years and 30 years, | 10 |
| 1 day, | 0 | " 30 " " 40 " | 10 |
| 1 day and 30 days, | 8 | " 40 " " 50 " | 15 |
| Between 1 month and 6 months, | 18 | " 50 " " 60 " | 8 |
| " 6 months and 12 " | 8 | " 60 " " 70 " | 5 |
| " 1 year " 2 years, | 6 | " 70 " " 80 " | 7 |
| " 2 " " 5 " | 16 | " 80 " " 90 " | 0 |
| " 5 " " 10 " | 6 | " 90 " " 100 " | 0 |
| " 10 " " 15 " | 1 | " 100 " | 0 |
| " 15 " " 20 " | 6 | Unknown, | 1 |
| Total, | | | 128 |

NATIVITIES.

| | | | |
|---|---|---|---|
| American, | 85 | French, | 3 |
| German, | 17 | Holland, | 0 |
| Irish, | 18 | Swiss, | 1 |
| Canadian, | 0 | Prussian, | 0 |
| English, | 4 | Italian, | 0 |
| Scotch, | 0 | Country not given, | 0 |
| Total, | | | 128 |

